# Nitric Oxide Synthase Regulates Gut Microbiota Homeostasis by ERK-NF-κB Pathway in Shrimp

**DOI:** 10.3389/fimmu.2021.778098

**Published:** 2021-12-03

**Authors:** Pan-Pan Hong, Xiao-Xu Zhu, Wen-Jie Yuan, Guo-Juan Niu, Jin-Xing Wang

**Affiliations:** Shandong Provincial Key Laboratory of Animal Cell and Developmental Biology, School of Life Sciences, Shandong University, Qingdao, China

**Keywords:** nitric oxide, *Marsupenaeus japonicus*, extracellular regulated protein kinase, antimicrobial peptide, microbiota

## Abstract

The gut microbiota is a complex group of microorganisms that is not only closely related to intestinal immunity but also affects the whole immune system of the body. Antimicrobial peptides and reactive oxygen species participate in the regulation of gut microbiota homeostasis in invertebrates. However, it is unclear whether nitric oxide, as a key mediator of immunity that plays important roles in antipathogen activity and immune regulation, participates in the regulation of gut microbiota homeostasis. In this study, we identified a nitric oxide synthase responsible for NO production in the shrimp *Marsupenaeus japonicus*. The expression of *Nos* and the NO concentration in the gastrointestinal tract were increased significantly in shrimp orally infected with *Vibrio anguillarum*. After RNA interference of *Nos* or treatment with an inhibitor of NOS, L-NMMA, NO production decreased and the gut bacterial load increased significantly in shrimp. Treatment with the NO donor, sodium nitroprusside, increased the NO level and reduced the bacterial load significantly in the shrimp gastrointestinal tract. Mechanistically, *V. anguillarum* infection increased NO level *via* upregulation of NOS and induced phosphorylation of ERK. The activated ERK phosphorylated the NF-κB-like transcription factor, dorsal, and caused nuclear translocation of dorsal to increase expression of antimicrobial peptides (AMPs) responsible for bacterial clearance. In summary, as a signaling molecule, NOS-produced NO regulates intestinal microbiota homeostasis by promoting AMP expression against infected pathogens *via* the ERK-dorsal pathway in shrimp.

## Introduction

Invertebrates lack adaptive immunity; therefore, they resist pathogens primarily *via* innate immunity, including cellular and humoral immunity. In humoral immunity, several immune effectors against pathogens have been identified in invertebrates, such as antimicrobial peptides (AMPs), melanin, and reactive oxygen species (1, 2). Now, a highly reactive and unstable free-radical gas, nitric oxide (NO) has been discovered as another effector in response to pathogen infection in several species of invertebrates (3). Several studies have demonstrated the direct toxicity of inducible NO to a number of pathogens, including viruses, bacteria, fungi, and parasites (4).

NO is synthesized through the oxidation of l-arginine to l-citrulline, mediated by the enzyme, nitric oxide synthase (NOS), in which is accompanied by the production of NO (5–7). Mammals have three types of NOS. Neuronal NOS (nNOS, also known as NOS1) exists in neurons, while endothelial NOS (eNOS, or NOS3) is distributed in endothelial cells, and both show Ca^2+^/calmodulin-dependent constitutive expression, thus they are also designated as constitutive NOS (cNOS) (8). nNOS and eNOS are now recognized to have a wider cell and tissue distribution, and to be also regulated by cytokines, microbial products, and hormones (9). The other type, inducible NOS (iNOS, or NOS2), whose expression is independent of Ca^2+^/calmodulin, is distributed in various cell types and can produce large amounts of NO rapidly after immune stimulation (10). However, to date, only a single NOS has been found in each invertebrate species, which has constitutive and/or inducible characteristics (11, 12). A NOS has also been identified in shrimp, *Marsupenaeus japonicus* (13), but its property and function need to be clarified.

NOS and its product NO play important roles in development, immunity, and inflammatory systems (14, 15). It has been reported that different NOS isoforms in vertebrates play the constitutive (signaling) and inducible (toxic to pathogens) roles against pathogen infection (3). NOS can be induced by pathogens, lipopolysaccharide (LPS), and certain immune-related factor, such as IFN-γ and interleukin 1 beta (IL-1β) (9, 16, 17). The expression of iNOS is positively regulated by the protein kinase C (PKC) signaling pathway in macrophages (18). In human skin cells, challenging with *Staphylococcus aureus* increases the expression of IL-33, which stimulates the suppression of tumorigenicity 2 (ST2, the IL-33 receptor)-protein kinase B (AKT)-β-catenin pathway to induce iNOS expression (19). In turbot fish, after the challenge with myxozoanparasite, *Enteromyxum scophthalmi*, a noticeable rise of iNOS expression is found in the intestines and lymphohematopoietic organs (20). NO also possesses the function of direct elimination of intracellular pathogens (21). In invertebrates, NOS was firstly characterized in several insects (12, 22), and NOS, along with its product, NO have been found to have a role in the induction of the invertebrate cellular and humoral immune responses (3, 23). NOS, for example, is identified as a downstream effector of the Janus kinase (JAK)/signal transducer and activator of transcription (STAT) pathway and exerts immunity against parasites in mosquitoes (24, 25). NO also promotes the expression of antimicrobial peptides (AMPs) *via* the immune deficiency (IMD) signaling pathway in *Drosophila* (11). NO can inhibit dengue virus replication partly through suppressing RNA-dependent RNA polymerase (26).

There are complex interactions between the microbiota and host gut cells in mammals. The microbiota performs functions that benefit host physiology. However, the microbiota is nonself, and the host must remain responsive to microbial breaches and invasion to control the microbiota homeostasis such that the symbiotic relationship is maintained (27). The epithelial Toll-like receptors in the mammalian gut are microbially induced proteins that play crucial functions in gut homeostasis *via* recognition of microbial motifs, enhancing the intestinal epithelial barrier, and shaping immune responses (28). The intimate interactions between the gut microbiota, epithelial cells, and immune cells, which are crucial for the maintenance of intestinal homeostasis in mammals, have been studied widely and reviewed recently (29). The intestinal epithelium can produce AMPs (30) and a secreted C-type lectin, RegIIIγ (31), to control the microbiota homeostasis. By contrast, the microbiota and its metabolites mediate the activation of the immune system in epithelial cells. For example, the symbiotic metabolite *n*-butyrate (a short-chain fatty acid) can reduce the expression level of LPS-induced proinflammatory mediators, such as several cytokines and NO, in intestines by inhibiting histone deacetylase; in this case, the decrease in intestinal immune sensitivity is conducive to maintaining the homeostasis of the intestinal flora (32). In invertebrates, there are increasing evidences that the midgut is an immune-competent organ, inducing immune-related signaling pathways or the expression of several immune effectors in response to pathogen infection and change of gut microbiota (33, 34). The interactions of gut microbiota and host immune system have been well reviewed, and immune effectors, such as AMPs and ROS are involved in the regulation of homeostasis of gut microbiota in *Drosophila* (35). In mosquito, *Anopheles stephensi*, heme peroxidase suppresses midgut immunity to support *Plasmodium* development. When the gene was silenced, the quantity of midgut bacteria decreased significantly *via* the activation of diverse immune pathways, including the induction of NOS expression (36). However, how NOS and its product NO exert their functions in gut microbiota homeostasis is mostly unclear in crustacean.

The shrimp gastrointestinal tract harbors an enormous number of microorganisms within the lumen (37). The constant contact with bacteria in the gut raises an important question: How does the shrimp intestinal immune system maintain tolerance to the gut microbiota and maintain its homeostasis? Our previous studies have found that ROS is involved in the gut microbiota homeostasis in shrimp (38, 39). In the present study, we identified a NOS in the shrimp *Marsupenaeus japonicus*, and found that NOS, and its product NO, were significantly increased in the gut of shrimp challenged by *Vibrio anguillarum*, a prevalent pathogen which causes serious economic losses in shrimp culture. After knockdown of *Nos*, the NO concentration decreased and the bacterial load increased significantly in the gut of shrimp. The possible mechanism by which NOS regulates gut microbiota homeostasis was analyzed.

## Materials and Methods

### Ethics Statement

The rabbit experiments for antibody preparation in the study were carried out in accordance with protocols approved by the Animal Care and Welfare Committee at Shandong University School of Life Sciences (SYDWLL-2021-53).

### Immune Challenge and Tissue Collection

The healthy shrimp *M. japonicus* (8–10 g) were purchased from a shrimp farm near Qingdao, Shandong Province, China. A shrimp culture system in the laboratory was used to cultivate the shrimp (40). For tissue distribution analysis, different organs, including the heart, hepatopancreas, gill, intestines, and stomach, were dissected from shrimp for protein and total RNA extraction. *Vibrio anguillarum* (10^8^ CFU) was fed into the mouthparts of shrimp as the immune challenge, except during the fluorescent immunocytochemistry assay. In the fluorescent immunocytochemistry assay, *V. anguillarum* (10^6^ CFU) was injected into the shrimp. PBS (140 mM NaCl, 2.7 mM KCl, 10 mM Na_2_HPO_4_, 1.8 mM KH_2_PO_4_, pH 7.4) was fed or injected into the control shrimp. Tissues were collected at different time points (3, 6, 12, 24, and 48 h) after bacterial infection for time-course expression analysis. The hemolymph was collected from the ventral sinus from three shrimp using a syringe containing an equal volume of precooled anticoagulant buffer (0.45 M NaCl, 10 mM KCl, 10 mM EDTA, and 10 mM HEPES, pH 7.45). The hemocytes were immediately harvested by centrifugation at 600×*g* for 8 min (4°C) from the experimental and control shrimp.

### RNA Extraction and cDNA Synthesis

Different organs (heart, hepatopancreas, gills, stomach, and intestines) and hemocytes from three shrimp were homogenized in the Trizol reagent (TransGen Biotech, Beijing, China), and total RNA was extracted according to the manufacturer’s protocol. cDNA was synthesized using 5 μg of the total RNA *via* a SMART cDNA synthesis kit (Clontech, Beijing, China) following manufacturer’s instructions. The obtained cDNA was kept in a −20°C freezer until use.

### Bioinformatic Analysis

The sequence of *Nos* was obtained from transcriptome sequencing in our laboratory. The amino acid sequence was predicted using the ExPASy tool (http://www.expasy.org). The online BLAST program (http://blast.ncbi.nlm.nih.gov/) was used to determine the similarity of NOS with other NOS proteins. The Simple Modular Architecture Research Tool (SMART) (http://smart.embl-heidelberg.de/) was employed to predict the domain architecture of NOS. Sequence alignment of *Nos* with other *Nos* genes was performed using MEGA 5.0 and GeneDoc software (http://www.flu.org.cn/en/download-47.html). The phylogenetic tree of NOS proteins was constructed using MEGA 5.0 with the neighbor-joining method (41).

### Semiquantitative Reverse Transcription PCR and Quantitative Real-Time PCR

Semiquantitative reverse transcription PCR (RT-PCR) was performed with cDNA obtained from different tissues as the template using primers *Nos*-RT-F and *Nos*-RT-R (**Table 1**) for tissue distribution analysis. The PCR reaction protocol was as follows: 94°C for 3 min; 94°C for 30 s, 54°C for 30 s, 72°C for 30 s (35 cycles); and 72°C for 10 min. The ATCB (*β-actin*) gene, as an internal control, was amplified using primers *β-actin*-RT-Fand *β-actin*-RT-R (**Table 1**).

**Table 1:** Sequences of primers used in this study.

Primers	Sequences (5′–3′)	GenBank accession no.	Amplicon size (bp)
cDNA synthesis			
SMART-F	TACGGCTGCGAGAAGACGACAGAAGGG		
Oligo anchor-R	GACCACGCGTATCGATGTCGACT16(A/C/G)		
RT-PCR			
*Nos*-RT-F	CACGAGCCCTTGATTTGTA	AB485762	302
*Nos*-RT-R	TTCATCCCTCATCTGTAGCA		
*β-actin*-RT-F	GCATCATTCTCCATGTCGTCCCAGT	GU645235	240
*β-actin*-RT-R	TACGGCTGCGAGAAGACGACAGAA		
RNAi			
*Gfpi*-F	GCGTAATACGACTCACTATAGGTGGTCCCAATTCTCGTGGAAC		
*Gfpi*-R	GCGTAATACGACTCACTATAGGCTTGAAGTTGACCTTGATGCC		
*Nosi*-F	GCGTAATACGACTCACTATAGGCTCGTCCTATCCGCTAATG		
*Nosi*-R	GCGTAATACGACTCACTATAGGAAGCCGCTGCTCGTTCT		
AMP expression			
*Alf-a1*-RT-F	CTGGTCGGTTTCCTGGTGGC	KU213607	219
*Alf-a1*-RT-R	CCAACCTGGGCACCACATACTG		
*Alf-b1*-RT-F	CGGTGGTGGCCCTGGTGGCACTCTTCG	KY627759	244
*Alf-b1*-RT-R	GACTGGCTGCGTGTGCTGGCTTCCCCTC		
*Alf-c1-*RT-F	CGCTTCAAGGGTCGGATGTG	KU213608	149
*Alf-c1-*RT-R	CGAGCCTCTTCCTCCGTGATG		
*Alf-c2*-RT-F	TCCTGGTGGTGGCAGTGGCT	KU160498	240
*Alf-c2*-RT-R	TGCGGGTCTCGGCTTCTCCT		
*Alf-d1*-RT-F	CTTTGGCGTGGAACAAGGTAGAGGAT	KU160499	243
*Alf-d1*-RT-R	GCTTTTTATTTTGGGGGTCACGCTGT		
*Alf-d2*-RT-F	CGCAGGCTTATGGAGGAC	MT977630	110
*Alf-d2*-RT-R	AGGTGACAGTGCCGAGGA		
*Alf-e1*-RT-F	TCCTAACCACGCAGTGCTTTGCTAATG	KY627760	216
*Alf-e1*-RT-R	GCTTTTCGGATTTGCCTTCGATGTTTG		
*Alf-e2*-RT-F	TGCCGTGTTCTCCTGCTTAT	KY627761	267
*Alf-e2*-RT-R	TTGGTGGGATTCGTGTGGT		
*CrusI1*-RT-F	TGCTCAGAACTCCCTCCACC	KU160502	173
*CrusI1*-RT-R	TTGAATCAGCCCATCGTCG		
*CrusI2*-RT-F	GCGTTTTCGTCTTCGTCCTG	MT977626	102
*CrusI2*-RT-R	AGTCCTTTCCGCCGTCACA		
*CrusI5*-RT-F	ATCGGCAAACCCGCAGTCTCTCT	KU213606	172
*CrusI5*-RT-R	CCGCTCTTCGTCGCAGCAGTAATAGT		
16S			
891F	TGGAGCATGTGGTTTAATTCGA		
1003R	TGCGGGACTTAACCCAACA		

Quantitative real-time PCR (qPCR) was used for time-course expression analysis of *Nos* and AMP genes in shrimp upon *V. anguillarum* challenge with *Nos* RT primers and antimicrobial peptide RT primers, and *β-actin* gene was used as an internal control (**Table 1**). After reverse transcription of RNA to cDNA, the cDNA was used as the template for a qPCR reaction comprising 95°C for 3 min; 40 cycles of 95°C for 15 s and 59°C for 50 s, followed by melting from 72°C to 95°C. The reaction mix contained 5 μl of 2× Premix Ex Taq, 1 μl of diluted cDNA, and 2 μl (1 mM) each of a pair of primers. Each experiment was performed in triplicate. The qPCR data were analyzed using the 2^−△△Ct^ method (42). Student’s *t*-test was used to assess whether the differences between the date were significant (*p* < 0.05) and One-way ANOVA was used for multiple comparisons.

### RNA Interference Assay

The template of *ds*RNA synthesis was obtained by RT-PCR amplification using primers *Nos*-Fi and *Nos*-Ri linked to the T7 promoter (**Table 1**). The *dsGfp* (green fluorescent protein) served as the control and was amplified using primers *Gfp*-Fi and *Gfp*-Ri (**Table 1**). DsRNA was synthesized using T7 RNA polymerase (Fermentas, Burlington, Canada) with an NTP mixture. For the RNA interference assay, the 50 µg of *dsRNA* was injected into the muscle of the penultimate segment of shrimp, and the same dose was injected again at 12 h. The efficiency of RNA interference was assessed at 48 h using qPCR. *V. anguillarum* was fed into mouthparts of shrimp at 48 h after the first injection.

### Bacterial Load Analysis *via* Bacterial Culture Assay

The number of bacteria was detected at 6 h after the interference experiment or NOS inhibitor (^N^G-monomethyl-l-arginine (L-NMMA)) injection. The stomach and intestines were dissected from three shrimp and homogenized in PBS. The obtained homogenate was diluted by 400- and 200-fold. Samples (50 μl) of the 400- or 200-fold diluted homogenate were placed on a plate containing Luria-Bertani (LB) medium for bacterial culture. The bacterial colonies were counted after incubation at 37°C overnight. The experiment was performed three times.

### Quantification of Gut Bacteria With qPCR Assay Based on 16S rRNA Gene

The method for bacterial load quantification using 16S rRNA genes mainly follows previous reports (43, 44). Briefly, for preparation of DNA sample of real-time PCR standards: Total microbiome DNA was extracted from shrimp gut samples using Genomic DNA Purufication Kit (Toyobo, Japan) and used for amplifying the target 16S rRNA genes by PCR *via* bacterial universal primers (891F and 1003R) (45) on a thermal Cycler T960 (Heal Force, Shanghai, China). The PCR product was then purified using SanPrep Column PCR Product Purification Kit (Sangon Biotech, Shanghai, China) and was quantified spectrophotometrically. A series of dilutions were then prepared, which were used as templates for quantitative real-time PCR with the primers (891F and 1003R). The cycle threshold (46) and the quantity of the template were used to make the standard curve for 16S rRNA gene quantification. The conditions of the qPCR assays of 16S rRNA genes were the same as those of the PCR described above. The copy number of 16S rRNA gene was calculated by following a previous method (43).

The gut samples from at least nine shrimp were collected and randomly divided to three samples of equal wet weight prior to DNA extraction. Total microbiome DNA was extracted using the above method for qPCR analysis with primers (891F and 1003R). The qPCR was performed to calculate 16S rRNA gene copies in shrimp using the standard curve. The qPCR procedure of 16S rRNA gene was the same as the ordinary qPCR described above, with the following modification, the PCR procedure consisted of an initial incubation at 95°C for 10 min, followed by 40 cycles of 95°C for 10 s and 60°C for 50 s, followed by a melting period from 65°C to 95°C. All the qPCRs were done in triplicate for both the standards and the microbial DNA samples.

### Protein Extraction and Western Blotting

The intestines from three shrimp were homogenized in radioimmunoprecipitation assay (RIPA) lysis buffer (Beyotime, Shanghai, China). The homogenate was centrifuged at 11,000×*g* for 10 min (4°C), and the supernatant was collected for further analysis. The supernatant was mixed with 3 × loading buffer (10% glycerin, 2% SDS, 0.1% bromophenol blue, 14.4 mM 2-mercaptoethanol, 50 mM Tris-HCl, pH 6.8) and incubated at 100°C for 10 min to denature the proteins fully. The sample was then separated using a 10% SDS-PAGE gel and transferred to a nitrocellulose membrane (0.45 μM) with transfer buffer (20% ethyl alcohol, 0.037% SDS, 25 mM Tris, 20 mM glycerin).

After blocking with 5% skimmed milk (in TBS, 10 mM Tris HCl, pH 7.5, 150 mM NaCl) for 1 h, the membrane was incubated with rabbit polyclonal antibodies (ERK antiserum, 1:200 dilution; β-actin antiserum, 1:500 dilution, phospho-ERK1-T202/Y204 + ERK2-T185/Y187, 1:5,000 dilution) for 1 h at room temperature, and then incubated at 4°C overnight. The ERK and β-actin antibodies were obtained by immunizing rabbits with recombinant proteins in the laboratory. Phospho-ERK1-T202/Y204 + ERK2-T185/Y187 rabbit antibodies were purchased from Abclone (AP0472, Wuhan, China). Histone-3 poly-clonal antibodies were purchased from Abclone (A2348, Wuhan, China). The membrane was washed three times with TBST (10 mM Tris HCl, pH 7.5, 150 mM NaCl, 0.02% Tween 20), 10 min each time, to remove nonspecifically bound antiserum. The membrane was then incubated with alkaline phosphatase-conjugated goat anti-rabbit IgG (ZB2308 ZSGB-Bio, Beijing, China) at room temperature for 3 h. The unbound antiserum was removed by washing three times with TBST for 10 min. TBS was then used to wash the membrane three times for 5 min each time. Thereafter, Substrate color developing solution, NBT (A610379, BBI)/5-bromo-4-chloro-3-indolyl phosphate (BCIP) (A610072, BBI) was added to visualize the signal in the dark at room temperature.

### Determination of Nitrite Concentration

NO is easily soluble in water and generates nitrite; therefore, the concentration of nitrite in shrimp tissues was determined using the Gress Reagent colorimetric method (47). The stomach and intestine tissues were obtained from three shrimp, weighed, and placed into a glass homogenizer containing nitric oxide detection reagents (Beyotime). After fully homogenizing the tissues on ice, the homogenate was centrifuged at 4°C, 13,000×*g* for 10 min, and the supernatant was retained. The supernatant was tested for its nitrite concentration using an NO assay kit (Beyotime). According to the manufacturer, the NaNO_2_ standard was used to construct a standard curve of NO.

### *In Vivo* Inhibitor Assay

For this experiment, the shrimp were divided into four groups. The L-NMMA (an inhibitor for NOS) solution (diluted in water) was injected into shrimp as pretreatment at the penultimate segment of the shrimp. The concentration of L-NMMA in shrimp of the different groups was 1.5, 3, and 6 µM. The other group of shrimp was injected with the same amount of ddH_2_O as a control. The pretreated shrimp were used in the subsequent experiments, i.e., detection of the NO concentration at 3 h; detection of the number of bacteria at 6 h; immune challenge as described above; and detection of the number of bacteria at 6 h.

### *In Vivo* NO Donor Injection Assay

The experimental shrimp were randomly divided into four groups. Different doses of sodium nitroprusside (SNP, a kind of NO donor) were injected into three groups of shrimp, with the final concentration in each group being 5, 10, and 20 μM per shrimp. The control group was injected with the same amount of ddH_2_O. The bacteria load at 6 h was then detected.

### Survival Rate Assay

We first performed an inhibitor toxicity test. Shrimp were randomly divided into four groups and each group contained 10 shrimp. Then, L-NMMA was injected into shrimp at different concentrations (0, 1.5, 3, and 6 μM). Shrimp injected with the same amount of ddH_2_O were used as the control. At 48 h postinhibitor treatment, shrimp survival was calculated. Next, 40 healthy shrimp were randomly divided into two groups; one group was injected with 3 μM L-NMMA and orally administrated with *V. anguillarum* (10^8^ CFU), and the control group was injected with ddH_2_O and then challenged by the same amount of bacteria. The dead shrimp were monitored every 12 h, and the survival rate were calculated using GraphPad Prism 5.0 software (GraphPad Inc, La Jolla, CA, USA).

Survival rate of NO-donor, SNP-treated shrimp challenged by *V. anguillarum* was also analyzed. The toxicity test of SNP was firstly carried out as the method described above using three different concentrations (5, 10, and 20 μM). Healthy shrimp were then randomly divided into two groups (25 shrimp each), and the survival rate of shrimp with 10 μM SNP injection following bacterial challenge was analyzed, using the same amount of ddH_2_O injection as the control.

### Fluorescent Immunocytochemistry Assay

First, a sterile syringe with 800 μl of prechilled anticoagulant buffer and 4% paraformaldehyde mixed solution (1:1) was used to collect an equal volume hemolymph from ventral sinus of three shrimp. After mixing, the solution was transferred to a precooled centrifuge tube and placed on ice for 10 min. The mixture was centrifuged at 600×*g* at 4°C for 5 min, and the supernatant was discarded. The pellet was gently suspended in 400 μl PBS, and then the suspension was dropped onto a cationic glass slide and incubated in the dark for 1 h. The unattached material was removed using six washes with PBS for 6 min each time. The glass slides were then treated with 0.2% Triton X-100 (in PBS) for 5 min to enhance the permeability of the cell membrane. Afterwards, the glass slides were washed with PBS for 5 min six times. The hemocytes were blocked in 3% BSA at 37°C for 30 min, and then incubated with antidorsal antibodies (1:20, diluted in 3% BSA) at 4°C overnight in the dark. The antidorsal antibodies were removed by washing six times with PBS. The cells on the slides were blocked with 3% BSA at 37°C for 5 min, and then incubated with Alexa fluor 488-conjugated anti-rabbit secondary antibodies (1:500 in 3% BSA) for 2 h at 37°C in the dark. The slides were washed with PBS six times for 5 min each and then incubated with DAPI (1:1,000 diluted in PBS) at room temperature for 10 min. After removing the DAPI by washing with PBS, the cells were covered with a coverslip and observed under an Olympus BX51 fluorescence microscope (Shinjuku-ku, Tokyo, Japan).

### Statistical Analysis

Data are presented as the mean ± the standard deviation (SD) of at least three replicates. Significance differences for paired comparisons were analyzed using Student’s *t*-test, asterisks in the figures indicate statistical significance (^*^ *p* < 0.05, ^**^ *p* < 0.01, and ^***^ *p* < 0.001). One-way ANOVA was used for multiple comparisons; the different lowercase letters indicate significant differences (*p* < 0.05) in the ANOVA analysis. Densitometry analyses of Western blot bands were based on three independent replicates using ImageJ software (National Institutes of Health, https://imagej.nih.gov/ij/download.html). Shrimp survival rate was calculated, and the survival curves are presented as Kaplan-Meier plots and the statistically using a log-rank test. All statistical analysis was produced using GraphPad 5.0 data view software.

## Results

### The Expression Level of *Nos* and NO Increased Significantly in Shrimp After *V. anguillarum* Challenge

**Figure 1:**
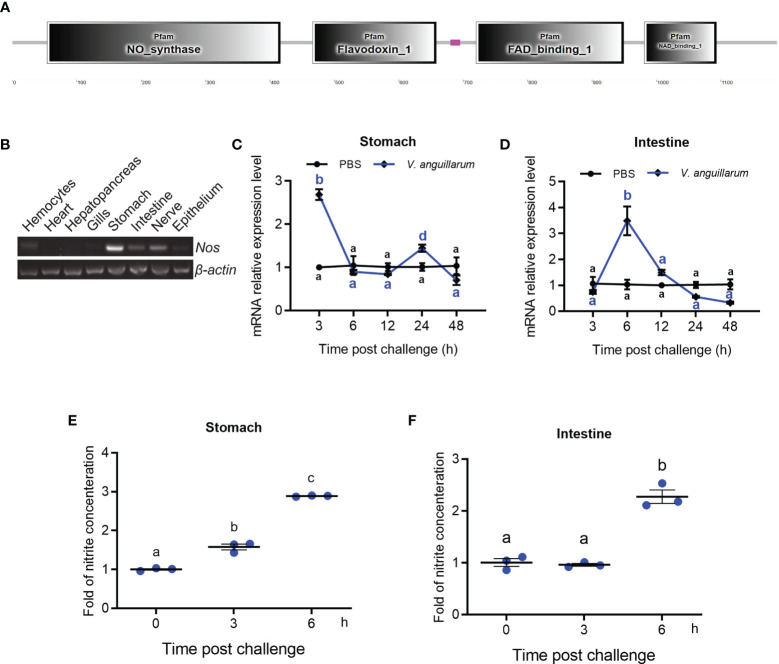
Tissue distribution and expression patterns of *Nos* in shrimp challenged by bacteria. **(A)** Domain architecture of shrimp NOS analyzed in the SMART web site (http://smart.embl-heidelberg.de). **(B)** Tissue distribution of *Nos* in hemocytes and different organs. **(C, D)** The expression patterns of *Nos* in the stomach **(C)** and intestines **(D)** in shrimp after *V. anguillarum* challenge, as analyzed using qRT-PCR; PBS injection was used as the control. **(E, F)** The concentration of nitrite in the stomach **(E)** and intestines **(F)** of shrimp after *V. anguillarum* challenge, as analyzed using the nitrite concentration determination method. Data represent the mean ± SD (*n* = 3). One-way ANOVA was used for multiple comparisons, the different lowercase letters indicate significant differences (*p* < 0.05) in the ANOVA analysis.

The cDNA sequence of *Nos* was obtained by intestinal transcriptome sequencing of *M*. *japonicus*. The domain architecture of the NOS comprises a NOS domain (AA 54–416), a flavodoxin–1 domain (AA 466–658), a FAD-binding–1 domain (AA 720–949), and an NAD-binding-1 domain (AA 981–1093) (**Figure 1A**). Phylogenetic analysis showed that the shrimp NOS was clustered with invertebrate NOS, which are distinct from vertebrate cNOS and iNOS (**Supplementary Figure S1**).

The tissue distribution of *Nos* in normal shrimp was analyzed using qPCR, which showed that *Nos* is mainly distributed in the gastrointestinal tract, including the stomach and intestines and the nervous system (**Figure 1B**). It could also be detected in hemocytes and the epithelium (**Figure 1B**). The mRNA expression patterns of *Nos* in the stomach and intestines were measured after bacterial challenge. In the stomach, *Nos* increased significantly at 3 and 24 h after bacterial challenge and then returned to normal levels (**Figure 1C**). In the intestines, significant upregulation of *Nos* occurred at 6 to 12 h after pathogen challenge (**Figure 1D**). We also tested the concentration of nitrite (to represent the NO concentration in solution) in the shrimp gastrointestinal tract after feeding with *V. anguillarum*. The results showed that the concentration of nitrite in the stomach and intestines increased significantly after 3 to 6 h of bacterial stimulation (**Figures 1E, F**). These results suggested that NOS might be involved in antibacterial immunity in the gastrointestinal tract of shrimp.

### The Nitrite Concentration Decreased and Bacterial Load Increased After Knockdown of *Nos* in Shrimp

**Figure 2:**
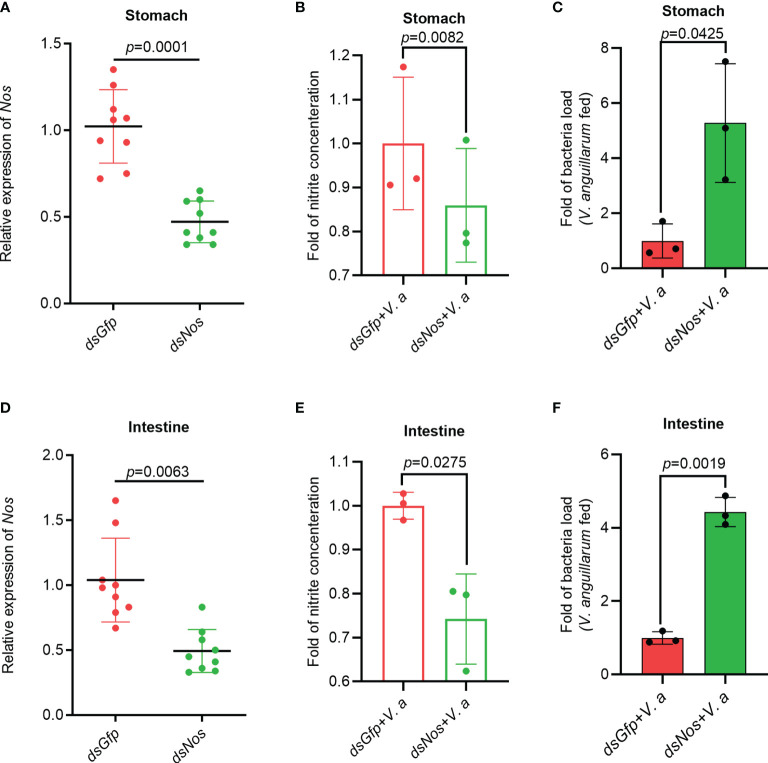
The Nitrite concentration decreased and the bacterial load increased in the gastrointestinal tract of shrimp after knockdown of *Nos*. **(A)** *Nos*-RNAi efficiency in the stomach analyzed using qPCR; *dsGfp* was used as the control. **(B)** The concentration of nitrite in the stomach of *Nos*-knockdown shrimp. **(C)** The bacterial load in the stomach of *Nos*-knockdown shrimp. **(D)** *Nos* RNAi efficiency in the intestines analyzed using qPCR; *dsGfp* was injected as the control. **(E)** The concentration of nitrite in intestines of *Nos*-knockdown shrimp. **(F)** The bacterial load in the intestines of *Nos*-knockdown shrimp. Significance differences were analyzed using Student’s t-test and significance was accepted at *p* < 0.05.

To analyze the function of NOS in the antibacterial response, *Nos* RNA interference (RNAi) was performed, and the nitrite concentration and bacterial load in the gastrointestinal tract were analyzed. After knockdown of *Nos* (**Figures 2A, D**), the shrimp were infected orally with *V. anguillarum*. The concentration of nitrite in the stomach and intestines decreased compared with that in the *dsGfp* injection group (**Figures 2B, E**). Correspondingly, the bacterial load in the tested tissues increased by about 5.3- and 4.2-fold in the *Nos-*knockdown group compared with that in the control group (**Figures 2C, F**). These results suggested that *Nos* plays a role in the homeostasis of the microbiota in the shrimp gastrointestinal tract.

### The Nitrite Concentration Decreased and Bacterial Loads Increased in Shrimp Treated With the NOS Inhibitor, L-NMMA

**Figure 3:**
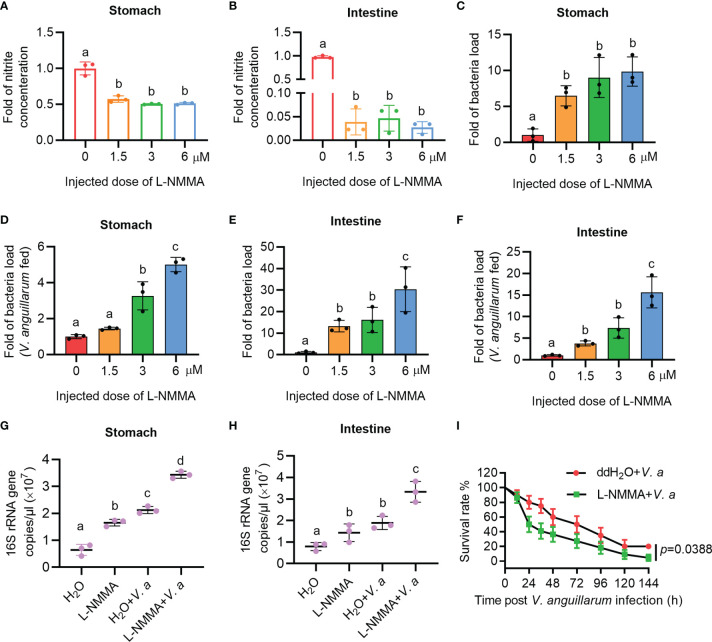
The nitrite level decreased and the bacterial load increased in the gastrointestinal track of shrimp after L-NMMA treatment. **(A, B)** Nitrite level in the stomach **(A)** and intestines **(B)** of L-NMMA-treated shrimp without bacterial infection, analyzed using the Gress Reagent colorimetric method. **(C)** The bacterial load in the stomach of L-NMMA-treated shrimp without bacterial infection. **(D)** The bacterial load in the stomach of L-NMMA- and *V. anguillarum* cotreated shrimp. **(E)** The bacterial load in the intestines of L-NMMA-treated shrimp without bacterial infection. **(F)** The bacterial load in the intestines of L-NMMA- and *V. anguillarum* cotreated shrimp. **(G)** The 16S rRNA gene copies in the stomach of shrimp injected with 3 μM L-NMMA before and after *V. anguillarum* infection. **(H)** The 16S rRNA gene copies in the intestines of shrimp injected with 3 μM L-NMMA before and after *V. anguillarum* infection. **(I)** Survival rate of shrimp injected with 3 μM L-NMMA following *V. anguillarum* infection; ddH_2_O + *V. anguillarum-*injected shrimp were used as the control. One-way ANOVA was used for multiple comparisons, the different lowercase letters indicate significant differences (*p* < 0.05) in the ANOVA analysis.

To further clarify the role of NOS in the gastrointestinal homeostasis of shrimp, L-NMMA was used to inhibit NOS activity in shrimp. The toxic effect of L-NMMA in shrimp was first analyzed by calculating the survival rate of shrimp after L-NMMA injection. Three different concentrations of L-NMMA (1.5, 3, and 6 μM/shrimp) were used in the experiments, following a previously reported method (48). The results showed that three concentrations of the inhibitor used in the experiment did not reduce the viability of the shrimp (**Supplementary Figure S2A**). The shrimp were then injected with three different concentrations of L-NMMA without further bacterial infection, and the nitrite concentration was analyzed in the shrimp gastrointestinal tract. The results showed that the concentrations of nitrite in gastrointestinal tract declined significantly in the inhibitor-injected shrimp in a dose-dependent manner (**Figures 3A, B**). The bacterial load increased significantly by 9.87 times in the stomach and 30.37 times in the intestines, of the 6μM/shrimp injection group compared with that in the control group (**Figures 3C, E**). Next, after injecting shrimp with L-NMMA, the shrimp were infected orally with *V. anguillarum*, and the bacterial number was analyzed. The results showed that the number of bacteria in the 6-μM L-NMMA injection group increased by 5.01 times in the stomach and 15.63 times in the intestines compared with that of the control group (**Figures 3D, F**). To confirm the above results, bacterial loads were also analyzed by detecting 16S rRNA gene copies *via* qPCR (**Supplementary Figure S3**). The results showed that after being injected with 3 μM L-NMMA, the 16S rRNA gene copies were increased significantly compared with the control group with or without bacterial infection, in the stomach (**Figure 3G**) and intestines (**Figure 3H**). The survival rate of shrimp injected with 3 μM L-NMMA per shrimp was also analyzed. The results showed that the survival rate of the shrimp was reduced significantly after treatment with L-NMMA and bacteria compared with the control (**Figure 3I**). These results suggested that NOS plays an important role against bacterial invasion and might be involved in the maintenance of microbiota homeostasis in the shrimp gastrointestinal tract.

### Injection of an NO-Donor Reduced the Number of Bacteria in the Gastrointestinal Tract

**Figure 4:**
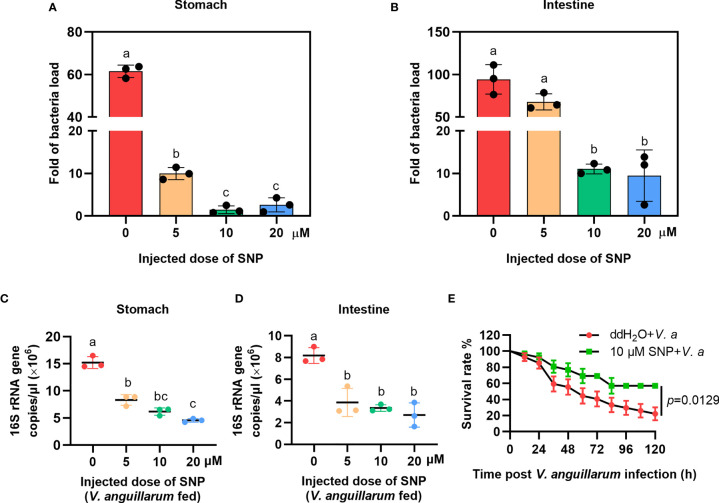
The bacterial load decreased in the gastrointestinal track of shrimp after SNP treatment. **(A)** The bacteria load in the stomach of SNP-treated shrimp. **(B)** The bacteria load in the intestines of SNP-treated shrimp. **(C)** The 16S rRNA gene copies in the stomach of SNP- and *V. anguillarum* cotreated shrimp. **(D)** The 16S rRNA gene copies in the intestines of SNP- and *V. anguillarum* cotreated shrimp. **(E)** Survival rate of shrimp injected with 10 μM SNP following *V. anguillarum* infection; ddH2O + *V. anguillarum*-injected shrimp were used as the control. One-way ANOVA was used for multiple comparisons, the different lowercase letters indicate significant differences (*p* < 0.05) in the ANOVA analysis.

To confirm the role of NO in shrimp gut microbiota homeostasis regulation, the NO-donor, SNP injection was performed and bacterial loads were analyzed. We first performed toxicity test of the shrimp treated with different concentrations of SNP (5, 10, and 20 μM); the results showed that the different doses of the inhibitor used in the experiment did not reduce the viability of the shrimp (**Supplementary Figure S2B**). To determine whether NO can resist bacterial infection, uninfected shrimp were injected with the NO-donor, SNP, and the number of bacteria was analyzed 6 h later. The results showed that the number of bacteria decreased after SNP treatment (**Figures 4A, B**). The bacterial loads were also analyzed by detecting the 16S rRNA gene copies in SNP-treated shrimp challenged by bacteria *via* qPCR. The results showed that the 16S rRNA genes were decreased significantly both in the stomach and intestines of the shrimp (**Figures 4C, D**). The survival rate of SNP-treated shrimp challenged by *V. anguillarum* was increased significantly after treatment with SNP compared with the control (**Figure 4E**). The results suggest that the NO-donor treatment can increase NO level and further induce the expression of antibacterial effectors against infected bacteria in shrimp.

### The Expression of AMPs in Gut of Shrimp Is Positively Regulated by NOS

**Figure 5:**
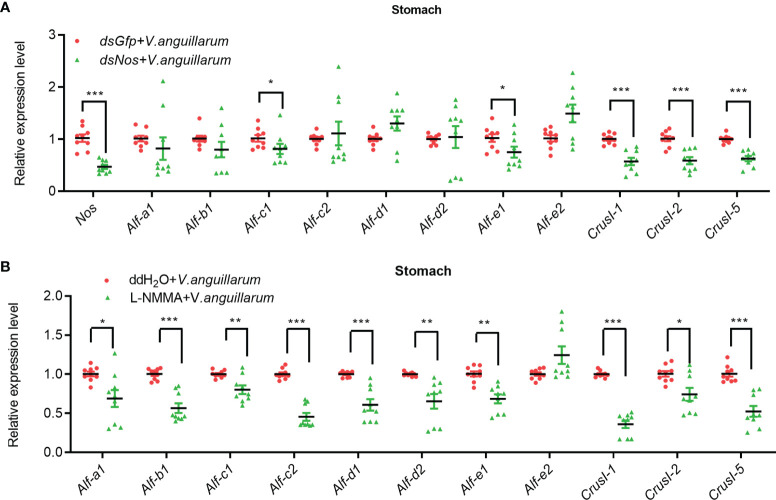
The expression of AMPs was inhibited in the stomach of shrimp after knockdown of *Nos* and L-NMMA treatment with bacterial challenge. **(A)** After knockdown of *Nos*, the mRNA expression levels of AMPs (ALF and CrusI families) in stomach of shrimp with bacterial challenge were detected by qPCR; *dsGfp* injection was used as the control. **(B)** The mRNA expression level of AMPs in stomach of L-NMMA-treated shrimp with bacterial challenge; ddH_2_O treatment was used as the control (^*^ *p* < 0.05, ^**^ *p* < 0.01, ^***^ *p* < 0.001).

To analyze the possible antibacterial mechanism of NOS in shrimp, we analyzed the mRNA expression of AMPs, including antilipopolysaccharide factors (ALFs) and Crustins in *Nos*-knockdown shrimp challenged by *V. anguilarum*. The results showed that the expression of *Alf-c1*, *e1*, and *Crus I-1*, *Crus I-2*, and *Crus I-5* was decreased significantly (**Figure 5A**). We also analyzed AMP expression in the stomach of *Nos*-RNAi-shrimp without bacterial challenge; the result showed that the expression levels of four *Alfs* (*Alf-c2*, *Alf-d2*, *Alf-e1* and *Alf-e2*), and two *CrusIs* (*CrusI-1* and *CrusI-5*) were also significantly decreased in the stomach of *Nos*-RNAi-shrimp (**Supplementary Figure S4A**). The AMP expression in the stomach of the L-NMMA-treated group challenged by bacteria was further analyzed; the results showed that the expression levels of seven *Alfs* and three *CrusIs* were decreased in the shrimp (**Figure 5B**). Similarly, the AMP expression in the stomach of the L-NMMA-treated shrimp without bacterial challenge was also analyzed, and similar results were obtained (**Supplementary Figure S4B**). All the results suggested that NOS might have constitutive expression in the stomach for NO production, and the NO in the stomach of shrimp with or without bacterial challenge acted as a signaling molecule to promote AMP expression.

**Figure 6:**
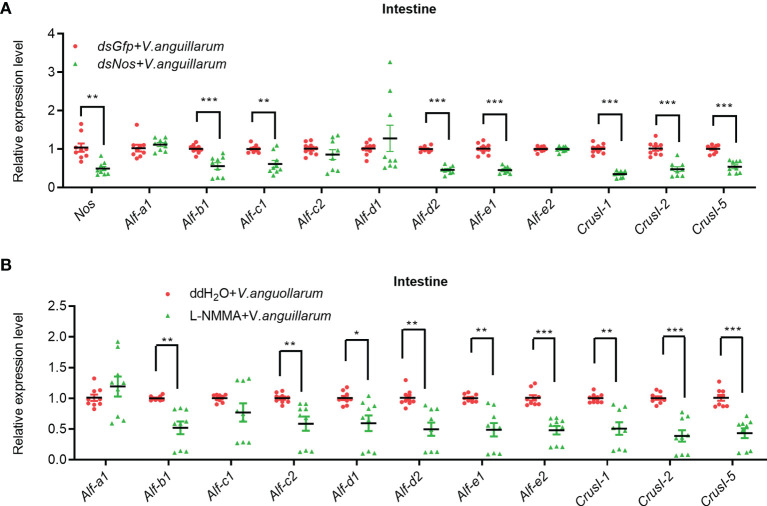
The expression of AMPs was inhibited in intestines of shrimp after knockdown of *Nos* or L-NMMA treatment with bacterial challenge. **(A)** After knockdown of *Nos*, the mRNA expression levels of AMPs were measured by qPCR in intestines of shrimp with bacterial challenge; *dsGfp* injection was used as the control. **(B)** The mRNA expression levels of AMPs in intestines of L-NMMA-treated shrimp with bacterial challenge detected by qPCR; ddH_2_O treatment was used as the control (^*^ *p* < 0.05, ^**^ *p* < 0.01, ^***^ *p* < 0.001).

The AMP expression in the intestines of *Nos*-RNAi- or L-NMMA-treated shrimp was further analyzed (**Figure 6**). The expression levels of four *Alfs* (*Alf-b1*, *Alf-c1*, *Alf-d2*, and *Alf-e1*) and three *CrusIs* (*CrusI-1*, *CrusI-2*, and *CrusI-5*) were significantly decreased in the intestines of *Nos*-RNAi-shrimp with bacterial challenge (**Figure 6A**). We also analyzed AMP expression in intestines of *Nos*-RNAi-shrimp without bacterial challenge; the result showed that the expression levels of several *Alfs* were decreased in the shrimp (**Supplementary Figure S5A**). In the L-NMMA-treated shrimp challenged by bacteria, expression levels of *Alf-b1*, *Alf-c2*, *Alf-d1*, *Alf-d2*, *Alf-e1*, and *Alf-e2*, *CrusI-1*, *CrusI-2*, and *CrusI-5* were declined significantly in the intestines of the shrimp compared with the control group (**Figure 6B**), and similar results were obtained in the intestines of the shrimp without bacterial challenge (**Supplementary Figure S5B**). The results suggested that NOS possessed constitutive expression in intestines for NO production, and the NO in intestines of the shrimp acted as a signaling molecule to promote AMP expression.

All above results suggested that the NOS possessed constitutive and inducible characteristics in gastrointestinal tract of shrimp, the NO generated by NOS in shrimp with or without bacterial challenge act as a signaling molecule to regulate AMP expression, and the AMPs expressed in the gastrointestinal tract were responsible for regulation of microbiota homeostasis in shrimp.

### The Increased NO Level Promotes ERK Phosphorylation in Shrimp Challenged by Bacteria

**Figure 7:**
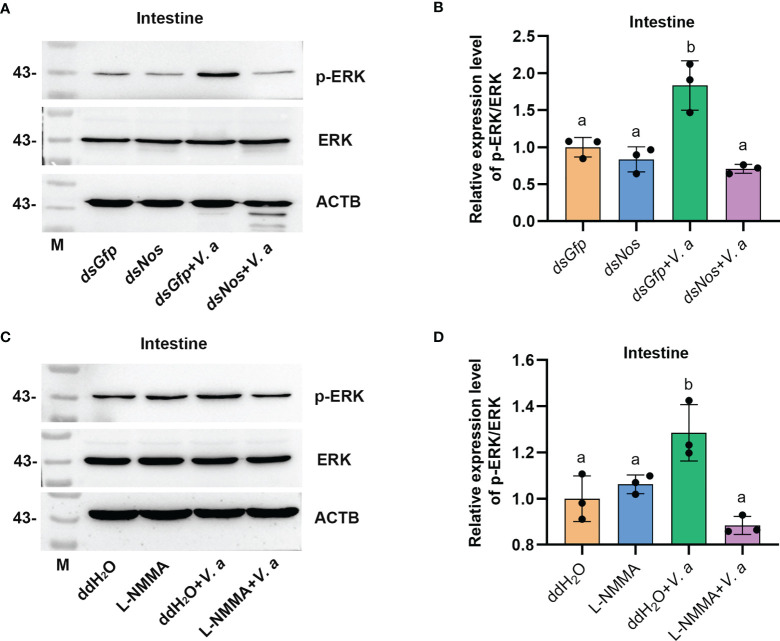
Increased NO level promotes ERK phosphorylation in shrimp challenged by *V. anguillarum*. **(A)** The level of p-ERK was analyzed by western blotting after *Nos* RNAi; *dsGfp* injection was used as the control, and ACTB (β-actin) and ERK were used as the loading control. **(B)** Statistical analysis of the data shown in **(A)** using ImageJ. Relative expression levels of ERK/ACTB were expressed as the mean ± SD, and the value of the control shrimp was set as 1. **(C)** The levels of p-ERK and ERK in shrimp after L-NMMA and *V. anguillarum* treatment; ddH_2_O was used as the control, and ACTB and ERK were used as the loading control. **(D)** Statistical analysis of the data shown in **(C)** using same method as in **(B)**. One-way ANOVA was used for multiple comparisons, the different lowercase letters indicate significant differences (*p* < 0.05) in the ANOVA analysis.

AMP expression is regulated by Toll, IMD, JAK-STAT pathways, or FOXO signaling in shrimp (34, 49, 50), and the transcription factors (including dorsal, relish, or STAT) of the pathways were phosphorylated and translocated into nucleus to promote AMP expression in response to pathogen infection. Bacterial infection upregulates NOS and promotes the activation of the NF-κB-like transcription factor in *Drosophila* (11). The level of phosphorylated ERK (p-ERK) increased after NO donor treatment in human breast MCF7 cells (51), and p-ERK promotes the translocation of the nuclear factor kappa B (NF-κB)-like transcription factor, dorsal, into the nucleus to regulate the expression of AMPs (52). To analyze how the NOS regulated AMP expression in shrimp, we firstly detected the ERK phosphorylation after knockdown of *Nos* or NOS inhibitor L-NMMA treatment in shrimp. The results showed that the level of p-ERK was significantly decreased in the intestines of *Nos*-RNAi shrimp (**Figures 7A, B**) and L-NMMA-treated shrimp (**Figures 7C, D**) after bacterial challenge for 1 h compared with that in the control group. However, no significant decrease was detected in the shrimp without bacterial challenge (**Figures 7A, D**). The results suggested that the increased NO level in shrimp challenged by bacteria is responsible for phosphorylation of ERK.

### Phosphorylated ERK Is Responsible for Dorsal Phosphorylation and Translocation Into the Nucleus in Shrimp

**Figure 8:**
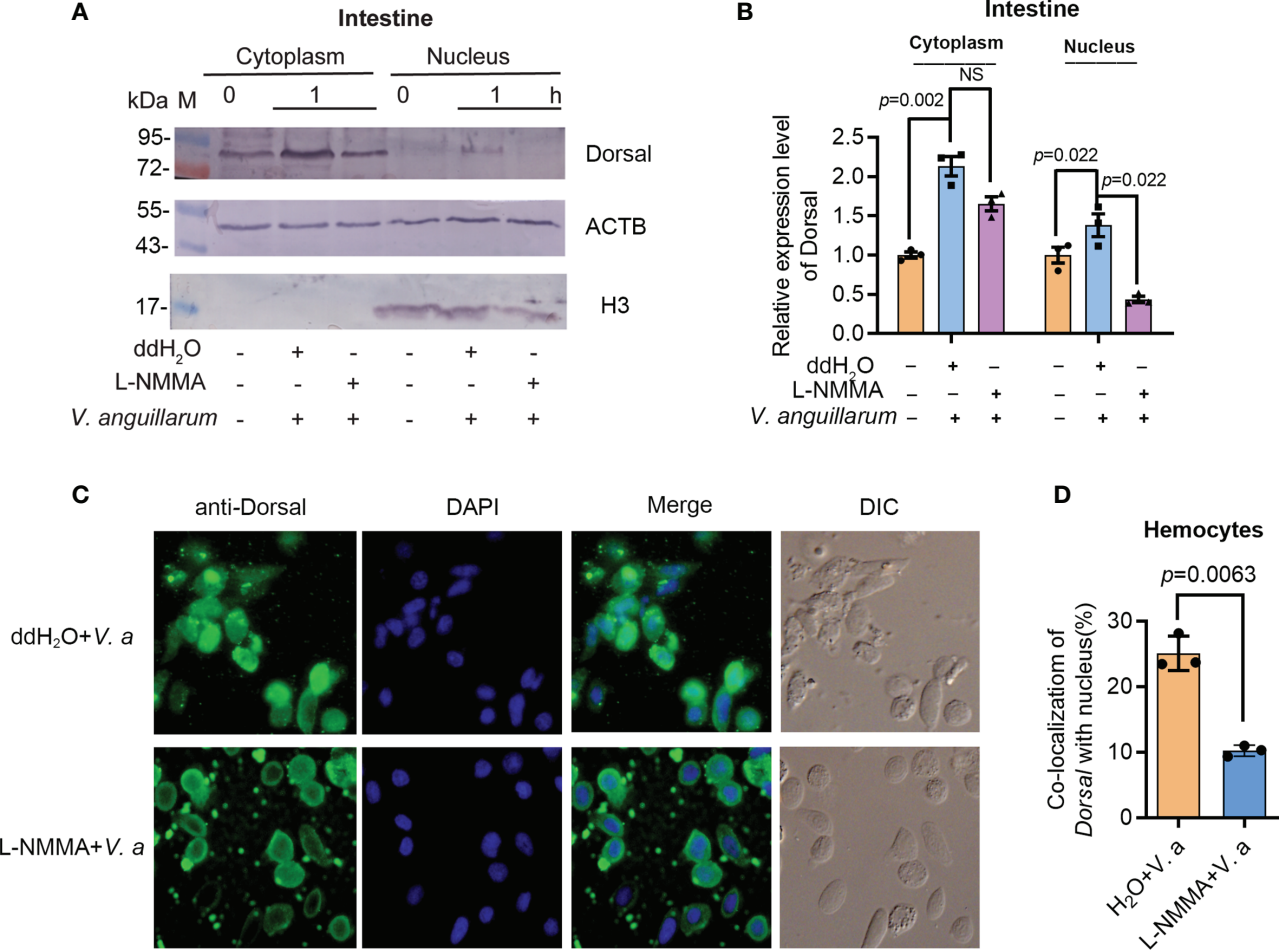
Activated ERK by NO signal is responsible for dorsal phosphorylation and translocation into the nucleus of hemocytes in shrimp. **(A)** The distribution of dorsal in the nucleus and cytoplasm of intestine cells after treatment with L-NMMA and *V. anguillarum*; ddH_2_O was used as the control; ACTB and histone 3 were used as loading controls. **(B)** Statistical analysis of the data from three experiments digitalized by ImageJ and statistical analyzed by Student’s *t*-test. **(C)** Subcellular distribution of dorsal in hemocytes of L-NMMA-treated shrimp challenged by *V. anguillarum* analyzed using fluorescent immunocytochemistry. The nuclei of hemocytes were stained using DAPI. **(D)** Statistic analysis of colocalization of dorsal with nucleus using WCIF ImageJ software. DIC, differential interference construct. Significance differences were analyzed using Student’s t-test and significant differences were accepted at *p* < 0.05. NS, no significant different.

To analyze if the phosphorylated ERK is related with dorsal phosphorylation and nuclear translocation, the subcellular distribution of dorsal was analyzed by Western blotting and fluorescent immunocytochemical assays. The translocation of dorsal into the nucleus decreased significantly in the intestines of L-NMMA-treated shrimp challenged by *V. anguillarum* compared with that in the control group (**Figures 8A, B**). Similar results were obtained in hemocytes of L-NMMA-treated shrimp challenged by bacteria analyzed by fluorescent immunocytochemical assay (**Figures 8C, D**). These results suggested that the activated ERK by NO signaling phosphorylated Dorsal and induced nuclear translocation of the transcription factor and then positively regulated expression of AMPs in shrimp.

## Discussion

In the present study, we found that the expression of *Nos* and the nitrite concentration (representing the NO content) were upregulated by bacterial invasion in the gut of shrimp. After restricting NOS function through RNAi or inhibitor treatment, in the gut, the NO levels decreased, the bacteria load increased, and the survival rate of shrimp declined significantly. The single NOS in shrimp had constitutive and inducible characteristics. The NO produced by the NOS functions as a signaling molecule to promote expression of AMPs responsible for homeostatic regulation of the gut microbiotas. To the best of our knowledge, NO participation in the regulation of gut microbiota homeostasis has not been reported before.

NO is an endogenously produced, short-lived gas in organisms. It serves as a key signaling molecule in various physiological processes or as an effector molecule to eliminate pathogens (53). NO in *Drosophila* and mosquitoes is produced by NOS under the stimulation of pathogens, such as bacteria and parasites, and can kill pathogens or activate downstream antibacterial reactions in systemic immunity (11, 24, 36). Glycosylphosphatidylinositol from *Plasmodium falciparum* upregulated the expression of NOS in immune cells and endothelial cells of *Aedes aegypti* and *Anopheles stephensi via* the insulin signaling pathway, and at the start of invasion, a large amount of NO would be toxic and kill the parasites (54, 55). After parasite infection, the midgut of *Anopheles stephensi* produces a large amount of NO to react with hemoglobin in enteric cavity, which created toxic substances such as heme ferric amide, ultimately affecting the growth of the malaria parasite (56). In the midgut of the tsetse fly, parasite Trypanosomes induce the host to produce NO to inhibit the colonization of the parasites (57). Several *in vitro* experiments had also proved that NO could kill a variety of pathogenic fungi and bacteria (58, 59). NO and nitrite are bacteriostatic agents due to its ability to inhibit protein activity of bacteria by NO-mediated *S*-nitrosylation of cysteine-containing proteins (3). In this study, we found that NOS and its product NO were upregulated in shrimp infected by bacteria, and the number of bacteria in the shrimp gastrointestinal tract increased significantly after knockdown of *Nos*. We also found that NO-donor application decreased bacterial loads significantly in shrimp, in the stay of bacterial challenge. The results suggest that NO works a signaling molecule or an effector molecule in the regulation of gut microbiota homeostasis.

In vertebrates, constitutive-derived low levels of NO produced by cNOS are involved in several physiological processes, including nervous transmission and vasodilatation. Inducible-derived high levels of NO produced by iNOS can exert beneficial effects on the host by acting as an antiviral, antibacterial, antifungal, antiparasitic, and tumoricidal agent or be detrimental to the host by acting as a cytotoxic effector (4). Similar to in other invertebrates, only one NOS was found in shrimp. The NOS is mainly distributed in the stomach, intestines, and nervous system and low expressed in hemocytes and the epithelium. The low level of NO produced by NOS in normal shrimp without bacterial challenge might maintain AMP expression at low level to keep microbiota homeostasis, suggesting the NOS shares characteristics of the constitutive NOS. On the other hand, NOS was inducible in the stomach and intestines of shrimp after oral bacterial infection, the increased NO produced by the NOS-induced AMP expression against bacterial challenge, suggesting that the NOS also shares the characteristics of inducible form of NOS. Therefore, the shrimp NOS might have constitutive and inducible characteristics of vertebrate cNOS and iNOS.

AMPs are present in many organisms, from invertebrates to vertebrates, and play a crucial role in innate immunity (60). The homeostasis of the microbiota in the human gastrointestinal tract is regulated by AMPs secreted by plasma cells and Paneth cells (61). NOS expression was induced in the midgut of *Drosophila* larvae-fed gram-negative pathogenic bacteria, and the resultant NO entered the fat body, where it activated the translocation of the transcription factor relish into the nucleus to promote the expression of the AMP diptericin (11, 62). Various antimicrobial peptide families such as penaeidins, crustins, ALFs, and stylicins have been identified in shrimp (49, 63). In our previous study, we found that activation of the JAK/STAT pathway increased the expression of *Alf-a1*, *Alf-c1*, *Alf-c2*, *CrusI-1*, and *CrusI-5* (64). The expression of *Alf-c2* and *CrusI-1* was regulated by the activation of Toll pathway (52). *Alf-b1*, *Alf-c1*, *Alf-c2, Alf-d2*, and *CrusI-7* are regulated by the IMD pathway (65). In the present study, the expression levels of several AMPs (*Alf-e1*, *CrusI-1*, *CrusI-2*, and *CrusI-5*) were regulated by NOS in shrimp (**Figures 5** and **6**). We further found that the activity of the transcription factor of Toll pathway, dorsal, was blocked after NOS inhibition. These results suggested that the NOS-regulated AMP expression might be mediated by the Toll pathway.

**Figure 9:**
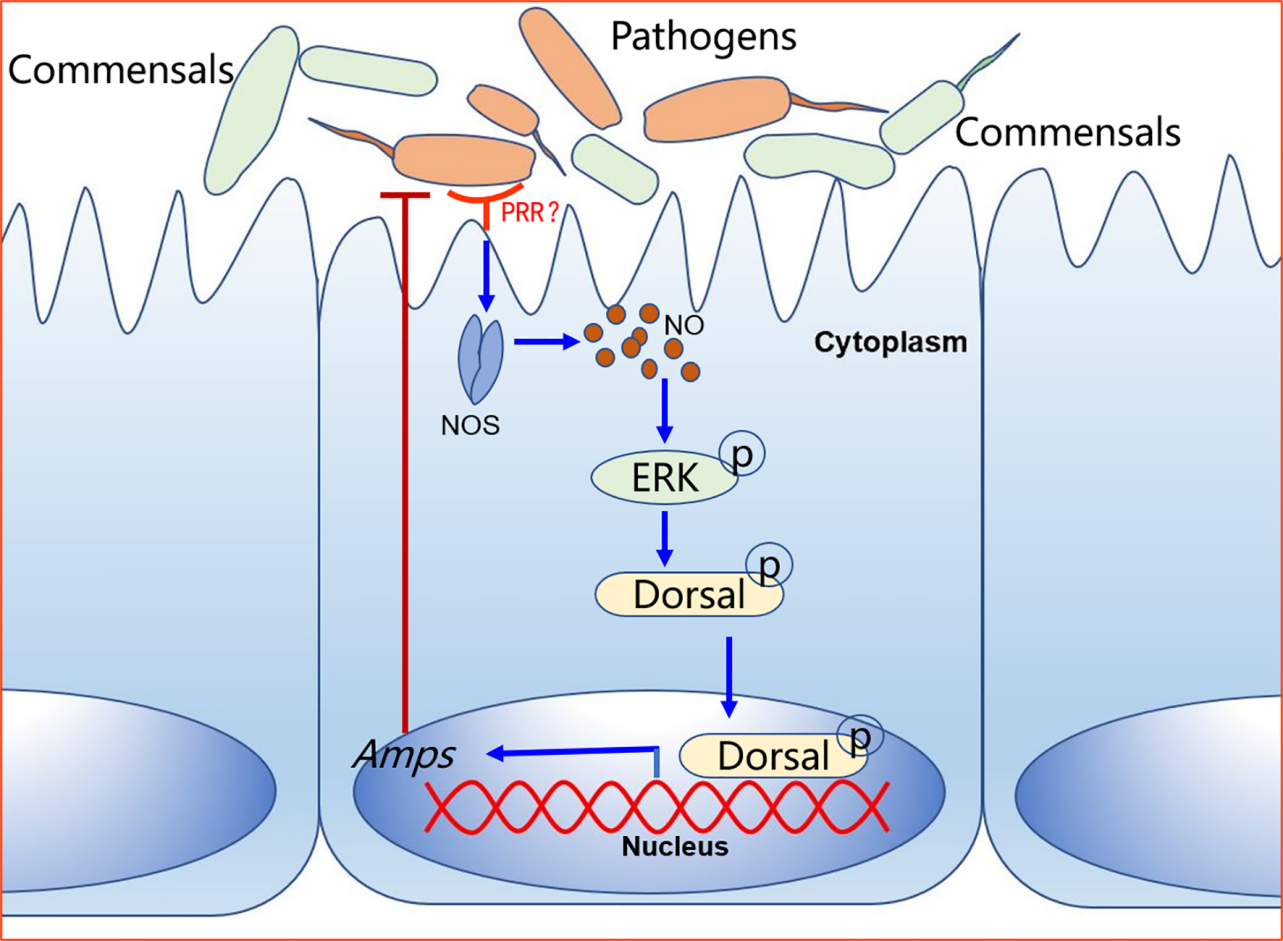
A model for NO signaling in intestinal antibacterial immunity after oral infection of shrimp *by V. anguillarum*. The pattern recognition receptor on the surface of intestinal cells recognizes pathogen-associated molecular patterns on pathogens and activates signaling. The host-pathogen interactions upregulate NOS in the intestine tissue to release NO, which activates a signaling cascade that leads to the expression of AMPs to defend against pathogens.

How does NOS regulate dorsal translocation into nucleus? In our previous study, we found that p-ERK promoted dorsal entry into the nucleus to upregulate the expression of AMPs (52). Therefore, we tested the level of p-ERK in intestines of NOS-inhibitor-treated shrimp and found that the level p-ERK was decreased in the shrimp stimulated by *V. anguillarum*. In addition, dorsal entry into the nucleus also decreased significantly in the intestines and hemocytes of the shrimp (**Figure 8**). These results indicated that the increased NO signal in shrimp challenged by bacteria might promote ERK activation, which would further phosphorylate and activate dorsal, promoting its translocation into the nucleus to increase the expression of AMPs in shrimp. Our results tentatively suggest the model shown in **Figure 9**. After sensing the infected pathogens by unknown pattern recognition receptors, NOS is activated to produce NO in gastrointestinal tract, which can induce ERK phosphorylation. Then, p-ERK promotes dorsal entry into the nucleus where it upregulates the expression of AMPs. The pathogenic bacteria in the intestines would be eliminated and the homeostasis of the gut microbiota would be maintained.

In the present study, we mainly focus on antibacterial immune response of gastrointestinal tract, especially the response on epithelial tissue of the gut because it serves a sentinel tissue in the case of natural infection. However, it should be noted that other tissues such as hemocytes might adopt this pathway upon systemic infection; other cells in gastrointestinal tract might also employ the pathway upon local infection. Therefore, we propose that host-pathogen interactions upregulate NOS in sentinel tissues and activate NO signaling that leads to the expression of AMPs against pathogens in systemic and local immune responses.

## Data Availability Statement

The datasets presented in this study can be found in online repositories. The names of the repository/repositories and accession number(s) can be found in the article/**Supplementary Material**.

## Ethics Statement

The animal study was reviewed and approved by the Animal Care and Welfare Committee at Shandong University School of Life Sciences (SYDWLL-2021-53).

## Author Contributions

J-XW conceived and conceptualized the work and strategy. P-PH, X-XZ, W-JY, and G-JN performed *in vitro* and *in vivo* experiments and analyzed and interpreted data. P-PH, X-XZ, W-JY, and J-XW wrote the manuscript. All authors supported the review of the manuscript. All authors contributed to the article and approved the submitted version.

## Funding

This work was supported by grants from the National Natural Science Foundation of China (Grant Nos. 31630084), and National Key Research and Development Program of China (Grant No. 2018YFD0900502), and NSFC (31930112).

## Conflict of Interest

The authors declare that the research was conducted in the absence of any commercial or financial relationships that could be construed as a potential conflict of interest.

## Publisher’s Note

All claims expressed in this article are solely those of the authors and do not necessarily represent those of their affiliated organizations, or those of the publisher, the editors and the reviewers. Any product that may be evaluated in this article, or claim that may be made by its manufacturer, is not guaranteed or endorsed by the publisher.
