# Supplementary material for: Nitric Oxide Synthase Regulates Gut Microbiota Homeostasis by ERK-NF-κB Pathway in Shrimp
Source: Front Immunol. 2021 Dec 3;12:778098. doi: 10.3389/fimmu.2021.778098 (PMC8678275; doi:10.3389/fimmu.2021.778098)
Supplement: Supplementary file 1 [file DataSheet_1.docx]

**Supplementary materials for**

**Nitric Oxide Synthase Regulates Gut Microbiota Homeostasis by ERK-NF-κB Pathway in Shrimp**

Pan-Pan Hong^1^, Xiao-Xu Zhu^1^, Wen-Jie Yuan, Guo-Juan Niu, Jin-Xing Wang*

Includes Five Figures:

Figure S1−S5.

**

**

**FIGURE S1.** Phylogenetic tree of NOSs from different species. A phylogenetic NJ tree was constructed by MEGA 5.0, the protein sequences of different species were obtained from GenBank. The repeatability of the result was checked by one thousand bootstraps. The length of branch was measured by the scale.


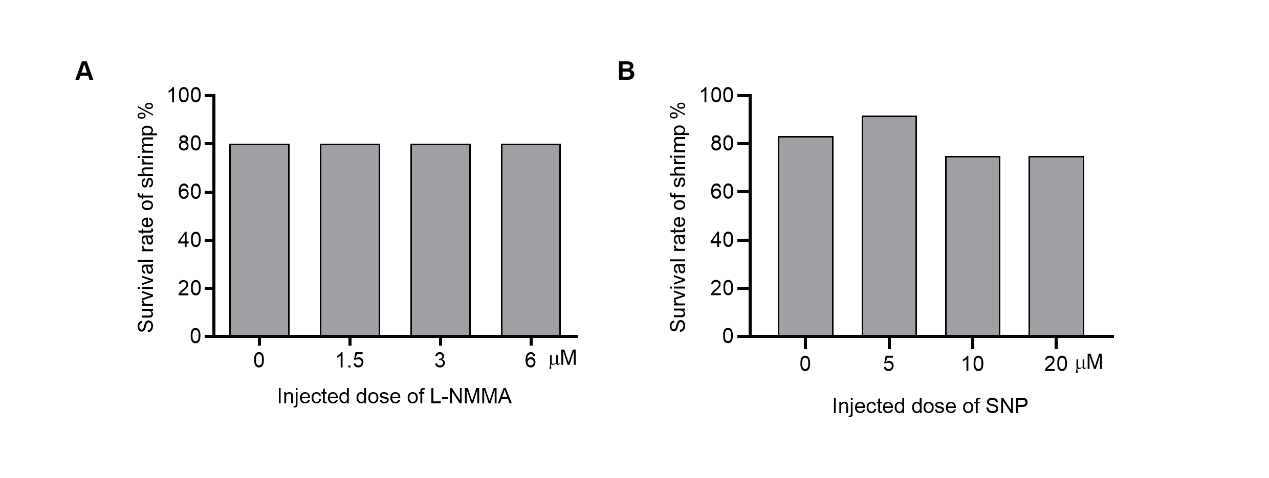


**FIGURE S2. (A)** Survival rate of shrimp at 48 h after injection of L-NMMA at different concentrations (1.5, 3, 6 μM/shrimp). **(B)** Survival rate of shrimp at 48 h after injection of SNP at different concentrations (5, 10, 20 μM/shrimp).


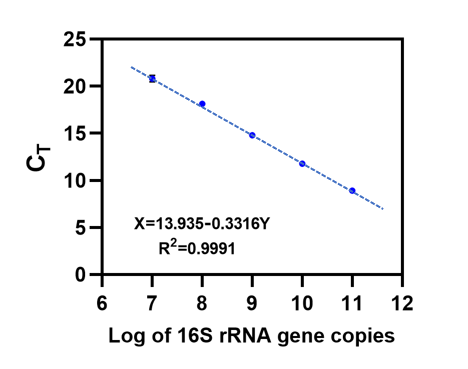


**FIGURE S3.** The standard curve of C_T_ value versus 16S rRNA gene copies number.


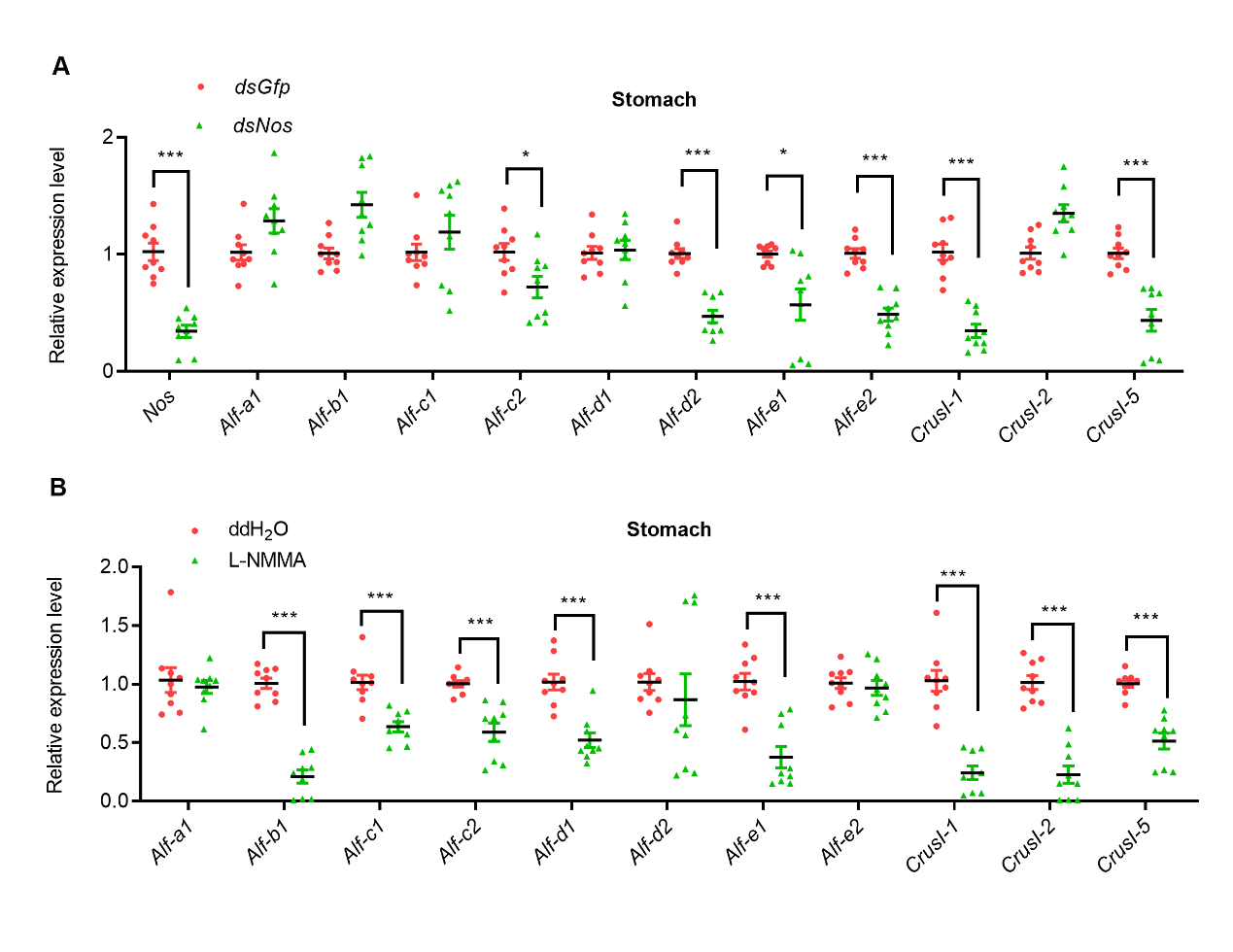


**FIGURE S4. The expression of AMPs was inhibited in stomach of shrimp after knockdown of *Nos* or L-NMMA treatment without bacterial challenge. (A)** After knockdown of *Nos*, the mRNA expression levels of AMPs were measured by qPCR in stomach of shrimp without bacterial challenge, *dsGfp* injection was used as the control. **(B)** The mRNA expression levels of AMPs in stomach of L-NMMA treated shrimp without bacterial challenge detected by qPCR, ddH_2_O treatment was used as the control (**P*< 0.05, ***P* <0.01, ****P* <0.001).


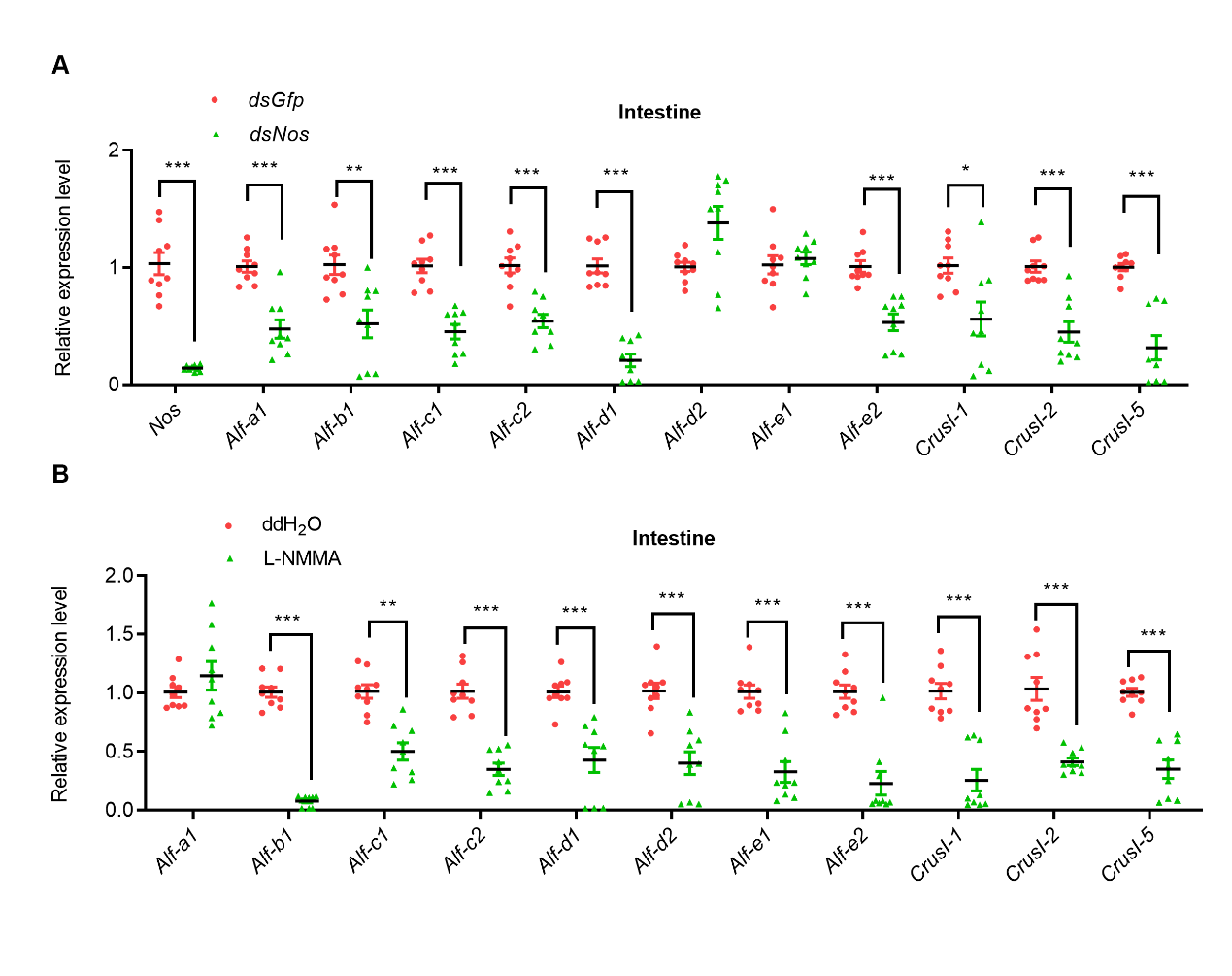


**FIGURE S5. The expression of AMPs was inhibited in intestine of shrimp after knockdown of *Nos* or L-NMMA treatment without bacterial challenge. (A)** After knockdown of *Nos*, the mRNA expression levels of AMPs were measured by qPCR in intestine of shrimp without bacterial challenge, *dsGfp* injection was used as the control. **(B)** The mRNA expression levels of AMPs in intestine of L-NMMA treated shrimp without bacterial challenge detected by qPCR, ddH_2_O treatment was used as the control (**P*< 0.05, ***P* <0.01, ****P* <0.001).
